# Relationship between Surface Properties and In Vitro Drug Release from a Compressed Matrix Containing an Amphiphilic Polymer Material

**DOI:** 10.3390/ph9030034

**Published:** 2016-06-24

**Authors:** Cristhian J. Yarce, Diego Pineda, Clara E. Correa, Constain H. Salamanca

**Affiliations:** Pharmaceutical Physical Chemistry Laboratory, Natura Research Group, Pharmaceutical Chemistry Program, Faculty of Natural Sciences, ICESI University, Cali 760031, Colombia; cjyarce@icesi.edu.co (C.J.Y.); d.pineda.qf@hotmail.com (D.P.); klaris02@gmail.com (C.E.C.)

**Keywords:** surface free energy, surface properties, drug release, amphiphilic polymer

## Abstract

The performance of compressed tablet drug delivery systems made using polymeric materials depend on multiple factors, such as surface properties like contact angle, surface free energy and water absorption rate, besides the release mechanisms driven by the kind of polymer used. Hence, it should be possible to establish a relationship between the surface properties and the drug release kinetics. Compressed tablets with different proportions of poly(maleic acid-*alt*-octadecene) potassium salt (0%, 10%, 20%, 30% and 40%) were prepared. Blends of a model drug (ampicillin trihydrate) and the polymer material were analyzed by DSC. The surface properties of the tablets were determined by the sessile drop method, while the surface energy was determined using the semi-empirical Young-Dupre, Neumann and OWRK models. The release profiles were determined simulating in vitro conditions (buffer solutions pH 1.2 and pH 7.4 with ionic strength of 1.5 M at 37 °C (310.15 K)). A kinetic analysis of the dissolution profiles using different models (zero order, first order, Higuchi and Korsmeyer-Peppas) was realized. The results showed a significant effect of the proportion of polymer in both the surface properties of the tablets and the dissolution release, indicating a relationship between the kinetic and thermodynamic properties.

## 1. Introduction

The last decades have been characterized by several advances in the chemistry and pharmacy fields, among which the development of new systems for controlled drug delivery represents an area of great interest [1,2,3,4]. These modified release methods depend on multiple factors, such as the shape and size of the tablets [5], the manufacturing process [6], the granulometric properties [7,8,9] and the physicochemical characteristics of the other compounds in the compressed blends, wherein polymer-based excipients are remarkable for their physicochemical properties and the diversity of their multiple applications [10,11,12,13,14,15,16]. Different kinds of natural, hemisynthetic and synthetic polymers have been used for this purpose, among which the copolymers derived from maleic anhydride have shown great potential as controlled drug release systems [10,17,18,19,20,21,22]. These kinds of copolymers have attracted great attention due to their biocompatibility characteristics, clearly defined structures and versatility to combine with other precursors leading to a wide variety of polymeric materials with multiple properties and applications [23,24]. Another notable property of these kinds of polymers is their ability to hydrolyze in aqueous media and form structures with two carboxylic acids that enhance the solubility in those media [25,26] while other functional groups in the polymer backbone can confer other properties to the material, turning them into potential excipients for controlled drug release systems.

One of these polymeric materials derived from maleic anhydride is the potassium salt of poly(maleic acid-*alt*-octadecene) referred to as PAM-18K, which is the subject of this study and has interesting surface properties, such as an ability to sharply decrease the surface tension of water [27] and form intra- and intermolecular hydrophobic pseudophases in aqueous media [17,28,29], Due to these properties and the fact that to date few studies have focused on establishing the relationships between surface and drug delivery properties [30,31], the aim of this work was to establish if there is a relationship between the release profiles of a zwitterionic model drug and the surface properties (contact angle, surface free energy and water absorption rate) of different compressed matrices containing different proportions (0%, 10%, 20%, 30% and 40%) of this polymer. In relation to the model drug selected, it was ampicillin trihydrate, which is a β-lactam moderately soluble in aqueous media whose solubility can vary with the pH of the medium [26]. Such variations in solubility depend on the structural features of the drug, which may exist as an anion, a cation or a neutral molecule in relation to its two pKa values of 2.65 and 7.24, corresponding to its carboxylic acid and amino groups, respectively. Thus its solubility is affected by the conditions of the dissolution medium, allowing one to establish the effect that the type of polymer material characteristics of solid matrices can have on the release and dissolution of zwittwerionic drugs in various aqueous media.

## 2. Theoretical Considerations

### 2.1. Contact Angle and Wettability

**Figure 1:**
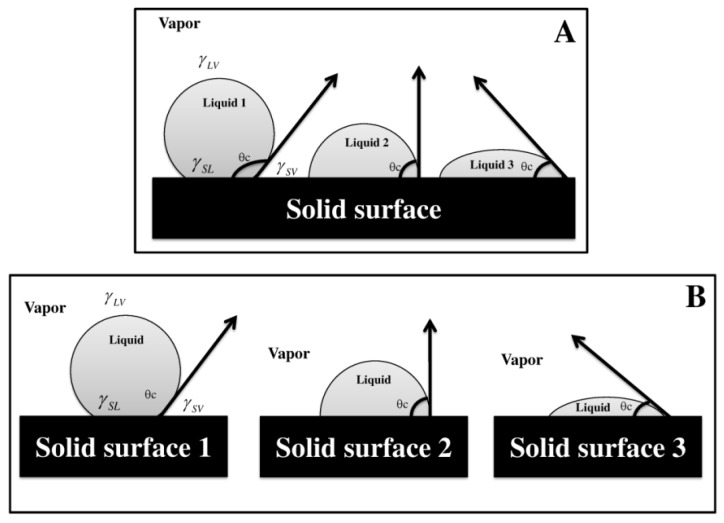
Graphical representation of the equilibrium states described by Young to define the contact angle. (**A**) Solid surface interacting with three different liquids; (**B**) Three solid surfaces interacting with the same liquid.

Thermodynamic descriptions of the phenomenon of liquid spreading on a solid surface described by Young [32,33], can be explained through a system as described in the following paragraphs. Figure 1 shows two interaction systems. The first case corresponds to a solid surface in contact with three different liquids, while the second case involves three different solid surfaces in contact with the same liquid. In both cases a three phase thermodynamic equilibrium system appears, since a drop is established between the liquid phase (*L*), the solid surface phase (*S*) and the gas phase (*V*). However, it is more convenient to express this equilibrium phenomenon arising from surface free energies expressed in terms of surface and interface tensions, such as:

(1) $$\gamma_{LV}\cos\theta_{c}=\gamma_{SV}-\gamma_{SL}$$

where *γ_SV_* and *γ_LV_* are the solid-vapor and liquid-vapor surface tension, respectively; *γ_SL_* represents the solid-liquid interfacial tension and *θ_c_* is the contact angle, which is established by a tangent to the liquid droplet and the solid surface in the area of intersection of the solid-liquid-vapor phases [32]. This experimental parameter is widely used to study the phenomenon of a liquid spreading on a solid surface, also known as “wetting” [34]. It has been stated that values below 90° for *θ_c_* indicate a spreading of the liquid on the solid surface and thus there is wettability; while values above 90° suggest a poor wettability of the liquid on the solid surface. For cases where the *θ_c_* is very close to 0°, the maximum spreading of liquid on the surface is obtained, corresponding to the highest wettability.

In the pharmaceutical field, the wettability corresponds to a very important phenomenon, as it can describe macroscopically the interaction that a solid dosage system (tablet) may have with different physiological fluids. Generally it has been described that when values for *θ_c_* < 90° are obtained, there are hydrophilic interactions between the solid surface and the dissolution medium, while values of 90° < *θ_c_* < 150° indicate that hydrophobic interactions are present, and when *θ_c_* > 150°, the nature of the interaction is of the super-hydrophobic type [35,36,37,38]. Therefore, depending on the degree of hydrophilic/hydrophobic interaction between the tablet surface and a physiological medium, a conditioning will exist for release of a drug present within the compressed matrix.

### 2.2. Surface Free Energy

The surface free energy (*G^s^*) or SFE is directly related to the surface tension between a liquid and a solid by the expression:

(2) $$\gamma ={\left(\frac{\partial G}{\partial A}\right)}_{P,T}=G^{S}$$

In the case of liquid systems the SFE is equivalent to surface tension (*γ_LV_*), which can be determined directly by several experimental methods such as the Du Nouy ring [39,40,41], the Wilhelmy plate [42,43] and the capillary rise [44,45]. However, in the case of solid systems, SFE (*γ_SV_*) cannot be determined directly as for liquids and is necessary to establish indirect relationships between the contact angle *θ_c_* and surface tensions. To realize this, different models have been established, such as the Young-Dupre [32,33], Neumann [32,46,47,48], OWRK [49,50], Wu [51] and Van Oss [48,52] ones.

The Young-Dupre model is one of the simplest and is related to a balance between two types of interactions that are generated in the solid-liquid system. The first of them corresponds to the cohesive interactions, which are produced by the solid and liquid in a pure state; while the second type corresponds to adhesion, which is given by interactions between the solid and the liquid. Depending on the balance between these two kinds of cohesive and adhesive interactions, contact angles with different values are obtained. This interfacial phenomenon is related to the work of adhesion (*W_adh_*), which is defined in terms of surface and interface tensions as:

(3) $$W_{adh}=\gamma_{LV}+\gamma_{SV}-\gamma_{SL}$$

where *γ_LV_, γ_SV_ y γ_SL_* correspond to the previously described parameters. This way, when Equations (1) and (2) are related, the Young-Dupre model is obtained [32,33], defined as:

(4) $$W_{adh}=\gamma_{LV}(Cos\theta_{c}+1)$$

This equation determines the work of adhesion (*W_adh_*), commonly dominated surface free energy, SFE, given between a solid and a liquid. The advantage of this model is that it allows one to acquire the SFE from two experimental parameters, such as the surface tension of the test liquid (*γ_LV_*) and *θ_c_* angle. Thus, the Young-Dupre equation allows one to obtain information about energy issues in the solid-liquid interface area, establishing a relationship between the contact angle *θ_c_* and the wettability phenomenon.

The Neumann model, which is known as the state equation [32,46] corresponds to a semi-empirical model derived from Young’s equation. In this model a correction factor termed *β* is included, which is related to the deformation of the liquid drop associated to gravity when the contact angle measurement is carried out. This model is defined as:

(5) $$\cos\theta_{c}=-1+2\sqrt{\frac{\gamma_{SV}}{\gamma_{LV}}}\times e^{-\beta {\left(\gamma_{LV}-\gamma_{SV}\right)}^{2}}$$

where *β* is a value of 1247 × 10^−4^ (m^2^/mJ)^2^ and *θ_c_, γ_SV_* and *γ_LV_* correspond to the same previously defined values. This model only takes into consideration the nature of the liquid phase in contact with the solid surface, so it is known as one-component model, since it is only necessary to evaluate a single liquid phase.

On the other hand, the Owens-Wendt-Rabel-Käelbe (OWRK) model [49,50], represents a more complex model, which discriminates the type and degree of interaction that occurs between the solid surface with the liquid drop. In this model it is possible to discriminate the surface free energy of the solid (*γ_SV_*) in terms of two kinds of interactions corresponding to the dispersive type (Van der Waals interactions) and the polar one type (dipole-dipole interactions and hydrogen bonds). The OWRK model is defined by a linear equation *y* = *mx* + *b*, as:

(6) $$\frac{\gamma_{LV}(\cos\theta +1)}{2{\left(\gamma_{LV}^{D}\right)}^{1/2}}={\left(\gamma_{SV}^{P}\right)}^{1/2}\frac{{\left(\gamma_{LV}^{P}\right)}^{1/2}}{{\left(\gamma_{LV}^{D}\right)}^{1/2}}+{\left(\gamma_{SV}^{D}\right)}^{1/2}$$

where $Y=\frac{\gamma_{LV}(\cos\theta +1)}{2{\left(\gamma_{LV}^{d}\right)}^{1/2}},\;m={\left(\gamma_{SV}^{p}\right)}^{1/2},\;X=\frac{{\left(\gamma_{LV}^{p}\right)}^{1/2}}{{\left(\gamma_{LV}^{d}\right)}^{1/2}},\;b={\left(\gamma_{SV}^{d}\right)}^{1/2}$.

In this equation, *p* and *d* correspond to the dispersive and polar contributions, respectively. Also *θ_c_*, *γ_SV_* and *γ_LV_* are the previously defined parameters. It should be noted that to evaluate this model is is necessary to use several test liquids, where the polarity therefore is increased.

The Wu model [51,53] is very similar to the OWRK model and requires the use of several reference liquids for evaluation and also allows discrimination to the SFE solid (*γ_SV_*) in terms of dispersive and polar contributions. The difference of this model is that it uses values harmonic means, while the OWRK model uses geometric means to obtain a better level of accuracy. The Wu model is defined as:

(7) $$\left[\frac{\gamma_{SV}^{d}\gamma_{LV}^{d}}{\left(\gamma_{SV}^{d}+\gamma_{LV}^{d}\right)}+\frac{\gamma_{SV}^{p}\gamma_{LV}^{p}}{\left(\gamma_{SV}^{p}+\gamma_{LV}^{p}\right)}\right]=0.25\gamma_{lv}(1+\cos\theta )$$

where *p* and *d*, *θ_c_*, *γ_SV_* and *γ_LV_* correspond to the previously defined parameters in the last equations.

Finally, the Van Oss-Chaudhury-Good model [48,52] like the OWRK and Wu models, also requires the use of several different liquids for evaluation and can discriminate between the contributions of dispersive (γ^d^) and polar interactions for SFE. The advantage of the Van Oss method is that it can achieve a higher degree of discrimination between the contributions of polar type, where it is possible to set the acid (γ^a^) and basic (γ^b^) contributions. Thus, the Van Oss model is useful in the case of solid surfaces formed by systems that can behave as potential electrolytes depending on the medium used. However, it should be mentioned that this model is very sensitive to small variations in the contact angles and the properties of liquids selected as probe, so one needs to be very careful in the handling when performing experiments. It should also be noted that there are discussions presenting this model as having mathematical conceptualization errors that lead to inappropriate conclusions [54]. The Van Oss model is defined as:

(8) $$\sqrt{\gamma_{SV}^{d}\gamma_{LV}^{d}}+\sqrt{\gamma_{SV}^{a}\gamma_{LV}^{b}}+\sqrt{\gamma_{SV}^{b}\gamma_{SV}^{a}}=0.5\gamma_{LV}(1+\cos\theta )$$

where *p* and *d* corresponds to the dispersive contribution, *a* and *b* correspond to the acid and basic polar contributions, respectively, and *θ_c_*, *γ_SV_* and *γ_LV_* correspond to the previously defined parameters in the last equations.

## 3. Materials and Methods

### 3.1. Materials

The precursor polymeric material was poly(maleic anhydride-*alt*-octadecene) with molecular weight about of 30–50 kDa (Sigma-Aldrich, St. Louis, MO, USA ) referred to as PAM-18 and was used as received. Ampicillin trihydrate (Fersinsa Gb) was provided by Tecnoquímicas Laboratories S.A. (Cali, Colombia) and was used as received. KOH (Merck KGaA, Darmstadt, Germany), KCl (Merck), KH_2_PO_4_ (Merck) and K_2_HPO_4_ (Merck) were also used as received. Type II water obtained from a purification system (Millipore Elix essential, Merck KGaA, Darmstadt, Germany), with values of pH and conductivity of 5.5 and 1 μS/cm respectively, was used to prepare the test solutions. For measurements of the contact angles were used as reference fluids: type I ultrapure water obtained from a purification system (Arium pro Sartorius Stedim biotechnology VF, Göttingen, Germany), with a conductivity value of 0.056 μS/cm. Isopropanol HPLC grade (LiChrosolv, Merck KGaA, Darmstadt, Germany) and ethylene glycol USP grade (Merck).

### 3.2. Obtaining and Characterization of PAM-18K Polymer

The PAM-18K polymer was obtained and characterized according to previously described methods [25,26]. To do this, the PAM-18 was reacted with an equimolar amount of KOH. The modification was carried out at room temperature for one hour with moderate agitation. Subsequently, the polymer solution was dialyzed using a 12 kD retention cellulose membrane (Sigma-Aldrich, St. Louis, MO, USA) until a constant conductivity value of about 5 μS/cm was reached. The polymer solution was then lyophilized in a frezeer (model FDU 1110, Eyela, Tokyo Rikakikai, Japan). Finally, a manual extrusion process to provide the solid polymeric material is carried out with 75 μm mesh (number 200). Structural characterization of the polymeric material was carried out in an infrared spectrometer (Nicolet 6700, Thermo Fisher Scientific, Waltham, MA, USA), where signals of the spectra of the precursor material PAM-18 and PAM-18K polymer were compared.

#### Preparation of Buffer Solutions

The buffer solutions with values of pH 1.2 and pH 7.4 and an ionic strength of 0.15 M, were prepared from mixtures of HCl/KCl, and KH_2_PO_4_/K_2_HPO_4_, respectively. KCl was used to adjust ionic strength.

### 3.3. Methods

#### 3.3.1. Granulometric Properties of Study Materials

External morphologies of the model drug and PAM-18K polymer were observed by scanning electron microscopy (SEMPro, PhenomWorld, Eindhoven, Nederland). The average granular diameter was obtained by tapping using a Ro-Tap RX-29 screening system (wsTyler, Mentor, OH USA). The percentage of compressibility was determined from Carr´s index, using a density meter (Logan Tap-2S). The flow degree was obtained by determining the angle of repose using a fixed funnel method.

#### 3.3.2. Thermal Characterization of Polymer-Drug Blends

Ampicillin trihydrate, PAM-18K polymer and their respective mixtures in proportions of 10%, 20%, 30% and 40% *w*/*w* of polymer relative to the drug, were analyzed in a Q2000 differential scanning calorimeter (DSC; TA Instruments, New Castle, DE, USA) calibrated with indium T_f_ = 155.78 °C ∆H_f_ = 28.71 J/g. DSC analysis were carried out using three heating-cooling cycles from −90 °C (183.15 K) to 200 °C (523.15 K) with a heating rate of 20 °C/min.

#### 3.3.3. Preparation of the Compressed Matrices

The tablets were made using a homemade tablet press with ¼ inch in diameter flat stainless steel punches. For each tablet 500 mg of ampicillin trihydrate in different proportions mixed with PAM-18K polymer, corresponding to 0%, 10%, 20%, 30% and 40% *w*/*w* were used. Three compression pressures of 200, 300 and 400 psi were applied for 10 s in each tableting. The hardness was determined using a durometer (Logan HDT-400), while the disintegration time was determined by an automated disintegrator (Logan USP DST-3) in type II water at 37 °C.

#### 3.3.4. Contact Angle Measurements

Determination of the static contact angle was carried out on the surfaces of each ampicillin trihydrate tablet with PAM-18K polymer, immediately after manufacture. The sessile drop method was used using a contact angle meter (OCA15EC Dataphysics Instruments, Filderstadt, Germany) with software driver (version 4.5.14 SCA20). Data capture was recorded on an IDS video camera, where the information from a range between 400 and 800 frames was taken as a reference point as a static angle. Moreover, the point capture of contact angle was defined as the reflected light of incident drop completely disappeared (about 1 s, since leaving the dispensing system). Drop volumes were in a range of 5 × 10^−3^ to 15 × 10^−3^ mL, and the liquid deposition fall was fixed to 1 cm for all assays. Each measurement was carried out at 22 ± 1 °C and 60% ± 5% of relative humidity. The contact angle was measured at least three times on different sites of the tablets surface. Each data reported is the average of triplicate measurements.

#### 3.3.5. Determination of Surface Free Energy (SFE)

Determination of the work of adhesion (*W_adh_*) given at the tablets interface of ampicillin trihydrate-PAM-18K with ultra-pure water was carried out using the Young-Dupre model, whereas the surface free energies of the tablets (γ_SV_) were determined from the OWRK and Neumann models. In the case of the Young-Dupre and Neumann models ultrapure water was used as reference liquid, while in the OWRK model propanol, ethylene glycol and water were used.

#### 3.3.6. Water Absorption Rate

Water absorption rates on the compressed tablets’ surface were determined by the change in the contact angle as a function of elapsed time or drop age on the solid surface. This measurement was carried out by the *non-static* contact angle tracking function provided by Dataphisycs software. The data were plotted as contact angle (°) vs. drop age (s). For the test, ampicillin trihydrate tablets with different proportions of PAM-18K polymer corresponding to 0%, 10%, 20%, 30% and 40% *w*/*w* were used. Every test was carried out until the point where the instrument failed to register more values of contact angle variation, either because all the liquid was absorbed or because a deformation took place on the solid surface.

#### 3.3.7. In vitro Dissolution Tests

Chemical stability assays for the ampicillin trihydrate under the study conditions were peformed due to remarkable degradability of this drug with respect to temperature and pH of the medium [55]. For this, a stress stability test was carried out under for 6 h at 37 °C. Ampicillin solutions of different concentrations were prepared in three media corresponding to ultra-pure water and pH 7.4 and pH 1.2 buffer solutions. Then consecutive samples were taken every 10 min and analyzed by HPLC with a LaChrom Ultra diode array detector (Hitachi-VWR, Radnor, PA, USA). The stability study results established that the maximum duration of the assay should not be longer than 1 h. In the case of ultra-pure water and buffer solution pH 7.4 is possible to use an UV spectrophotometry quantification methodology. However, in the case of buffer solution pH 1.2 it is necessary to use a HPLC methodology.

On the other hand, the dissolution test was carried out using the paddle method on a previously calibrated tester (apparatus II, Vision G2 Classic 6-Hanson, Chatsworth, CA, USA). The paddle speed was 100 rpm at a temperature of 37.0 °C ± 0.5 °C. The media volume of simulated gastric and plasma conditions (buffers pH 1.2 and pH 7.4 with ionic strength of 0.15 M, respectively) were 900 mL. Each dissolution test was carried out for 45 min, where a 5 mL sample was taken with replacement at predetermined time intervals. The samples were filtered through 0.45 μm filter. Determination of the ampicillin amount in ultra-pure water and buffer solution pH 7.4 was carried out by UV spectrophotometry at 256 nm at 37.0 °C (310.15 K) using a UV spectrophotometer (Shimadzu, Kyoto, Japan) coupled to temperature control system; whereas in pH 1.2 buffer a HPLC methodology was used under the same conditions described in the stability assay.

The data obtained from the in vitro dissolution profiles are reported as the average dissolution efficiency (DE) of the tablet [56]. This parameter is defined as the area under the dissolution curve (AUC) recorded at a particular time in relation to the rectangular area (R) described by 100% of dissolution at the same time. The efficiency of the solution can be calculated from:

(9) $$\mathrm{D.E}=\frac{\mathrm{AUC}}{R}\times 100\%=\frac{\int_{0}^{t}y\times dt}{y_{100}\times t}\times 100\%$$

where *y* is the dissolved drug percentage in a time *t*.

#### 3.3.8. Kinetic Study of the Model Drug Release

In an effort to investigate the kinetics and mechanisms that govern the process of model drug (ampicillin trihydrate) release from the tablets containing the PAM-18K polymer, the following kinetic models were applied:

(1) The zero order model: This model is widely used for pharmaceutical dosage systems that do not disintegrate and in which they have a very slow drug release. Furthermore, for this model it is assumed that the area of the tablet does not change significantly and material balance conditions are not formed. This model is expressed by the equation:
  (10) $$Q_{t}=Q_{0}+k_{0}t$$
  where *Q_t_* is the amount of dissolved drug at time *t*, *Q_0_* is the initial amount of drug in the solution (most of times *Q_0_* = 0) and *k_0_* corresponds to the constant of zero order release [57].
(2) The first-order model: This model is commonly used to describe the absorption and release of water soluble drugs from porous matrices. However it is difficult to contextualize this mechanism to a theoretical basis. This model can be expressed by the equation:
  (11) $$LogQ_{t}=LogQ_{0}-\frac{k_{1}}{2.303}t$$
  where *Q_t_* is the amount of dissolved drug at time *t*, *Q_0_* is the initial amount of drug in the solution and *k_1_* corresponds to the constant of first order release [56].
(3) The Higuchi model: This model is widely used to describe the release of soluble and sparingly soluble drugs in aqueous media, from various semi-solid and/or solid matrices according to the equation:
  (12) $$Q_{t}=k_{H}t^{1/2}$$
  where *k_H_* is the Higuchi dissolution constant, while *Q_t_* and *t* correspond to the same parameters described previously [58].
(4) The Korsmeyer–Peppas model: This is a generalized model of the Higuchi equation that allows one to explain drug delivery mechanisms where erosion and/or dissolution of the matrix occurs. This model has been widely used to describe the drug release from polymer systems. The related equation is:
  (13) $$\frac{M_{t}}{M_{\infty }}=k_{r}t^{n}$$
  where M_t_/M_∞_ corresponds to the fraction of drug released at time *t*; *k_r_* is the release constant which is characteristic for the polymer-drug interactions, while *n* is the diffusion exponent that is characteristic for the release mechanism. When *n* equals 0.5, the equation becomes equal to the Higuchi model, indicating that the release mechanism is of a Fickian type (case I), while values of *n* between 0.5 and 1.0 suggest that the release mechanism corresponds to an anomalous (non-Fickian) transport. Values of 1.0 indicate that the release mechanism is similar to a zero order release, while values of *n* greater than 1.0 (Super Case II transport), suggest a drug release process dependent of the relaxation of the polymer chains in the matrix, passing from a vitreous state (lower kinetic movement and increased potential energy) to a relaxed state rubber type (high kinetic movement and lower potential energy). For systems with a cylindrical matrix, the values of *n* are replaced by 0.45 instead of 0.5 and 0.89 instead of 1.0. The determination of *n* should be carried out only with a portion of drug release of 60% [56,59].

### 3.4. Data Processing and Analysis

The data were tabulated and analyzed using Microsoft Excel and Graph Pad Prism 6, respectively. The homogeneity of variance in the data was analyzed using Bartlett’s test. Statistical comparisons were made using an one-way ANOVA. The Bonferroni post-hoc test was used to determine significant differences between the two independent groups. A confidence level of 95% was adopted. Data are expressed as mean ± standard deviation.

## 4. Results and Discussion

### 4.1. Obtaining and Characterization of PAM-18K Polymer

The formation of the PAM-18K polymer was evidenced by a qualitative change in the solubility, which went from being a heterogeneous mixture to a completely homogeneous solution. This change is caused by the anhydride group opening in the precursor polymer PAM-18 that leads to the formation of two carboxylic acid groups, which are then converted to carboxylates. This transformation was shown by comparison of FTIR spectra between PAM-18 and PAM-18K, where the disappearance of the signal at 2358 cm^−1^ was observed in the PAM-18, corresponding to the opening of the bond C-O-C in the maleic anhydride substituent. Typical signals were also observed at 920 and 2848 cm^−1^ corresponding to the symmetric and asymmetric stretching of the CH bonds. More changes in the carbonyl signals at 1773 and 1704 cm^−1^ to 1706 and 1556 cm^−1^ were observed Finally, the appearance of a band at 3401 cm^−1^ was observed, indicating the presence of a hydroxyl group, from the formation of carboxylic acid groups indicating that the ionization process of PAM-18 and PAM-18K is not complete, as expected and widely reported [25,26].

### 4.2. Granulometric Properties of the Study Materials

**Figure 2:**
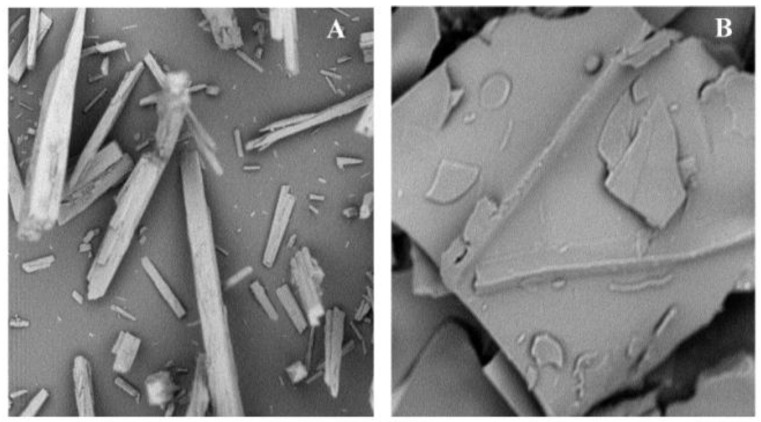
SEM photographs of: (**A**) ampicillin trihydrate (600×); (**B**) PAM-18K (2250×).

The external morphology of study materials corresponding to the model drug and polymeric material PAM-18K are shown in Figure 2, where needle and plaque morphologies are seen, respectively.

The average diameter results of the solid particles of ampicillin trihydrate and PAM-18K polymer obtained after the manual extrusion process were 53 and 75 µm, respectively. On the other hand, the compressibility percentage (Carr´s index) for ampicillin trihydrate was 7%, while the PAM-18K polymer one was 15%, indicating good compressibility properties. Regarding the value of repose angle for the trihydrate ampicillin it was found to be 44°, while for the PAM-18K polymer it was 36°, indicating a poor flow for both materials, which is consistent with the presented morphology of the study materials [8].

### 4.3. Thermal Characterization of Polymer-Drug Mixtures

**Figure 3:**
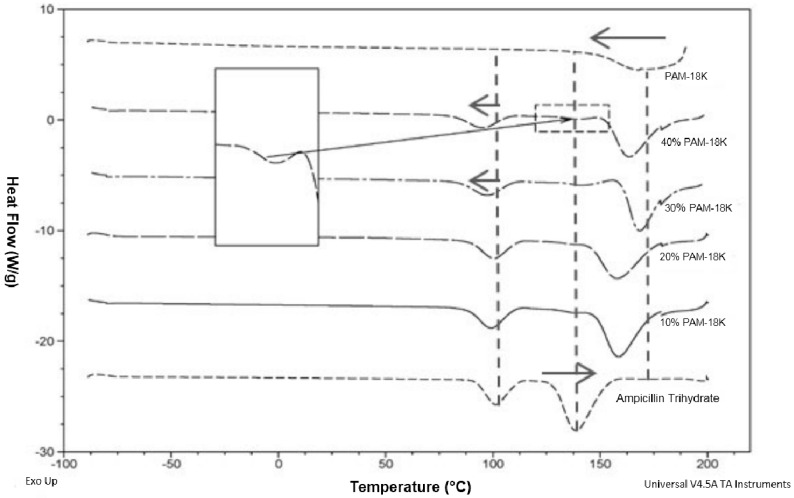
Thermograms of ampicillin trihydrate with PAM-18K polymer at different proportions.

Figure 3 shows a strong interaction between the PAM-18K polymeric material and the model drug ampicillin trihydrate.

The evidenced behavior is attributed to two phenomena: (1) a decrease in the thermal transition temperature of the PAM-18K polymer at about 175 °C and a higher thermal transition temperature of the model drug to 137.5 °C, which becomes stronger as the amount of polymer in the mixture increases; (2) appearance of a new thermal signal, which begins to increase in intensity and energy, directly related to the increase in the proportion of polymer in the blend. These observed phenomena are a clear manifestation of strong interactions between the polymer material and the model drug [60,61,62].

### 4.4. Preparation of the Compressed Matrices

The hardness and disintegration time results for a tablet of 500 mg of ampicillin trihydrate at different proportions of PAM-18K polymer, corresponding to 0%, 10%, 20%, 30% and 40% *w*/*w*, prepared under three different compression forces are summarized in Table 1.

The hardness and disintegration time results for ampicillin trihydrate with PAM-18K polymer tablets show that an increase in the compression leads to an increase of both parameters; which was expected because a higher degree of material compaction within the tablets is achieved [6]. However, it is noteworthy that for tablets with polymer proportions between 10% and 20% a faster disintegration occurs than for the ampicillin alone, whereas for proportions of polymer between 30% and 40%, the disintegration time is increased, suggesting an effect of the proportion of polymer within the PAM-18K tablets on disintegration. From these results, it was established as a condition for the development of tablets to apply a pressure of 300 psi for a period of 10 s using the ¼ inch flat punch.

### 4.5. Contact Angle Measurements (θ_c_)

**Figure 4:**
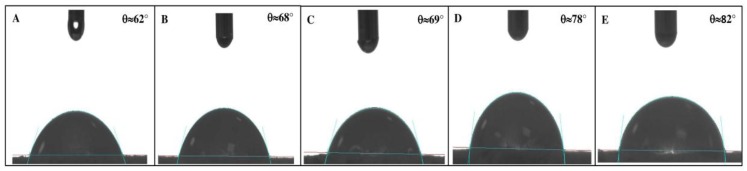
Contact angle variation between ultra-pure water and ampicillin trihydrate tablets at different PAM-18 K polymer proportions. (**A**) 0%; (**B**) 10%; (**C**) 20%; (**D**) 30%; (**E**) 40%.

In order to know how ampicillin trihydrate tablets interact with the PAM-18K polymer regarding the dissolution medium, a study of the surface properties of the tablets was carried out as described below. Initially, the effect of addition of ultra-pure water drops onto the tablet surface was evaluated, where a variation of *θ_c_* was found according the increase of the polymeric material incorporated within the tablets, as shown in Figure 4.

**Figure 5:**
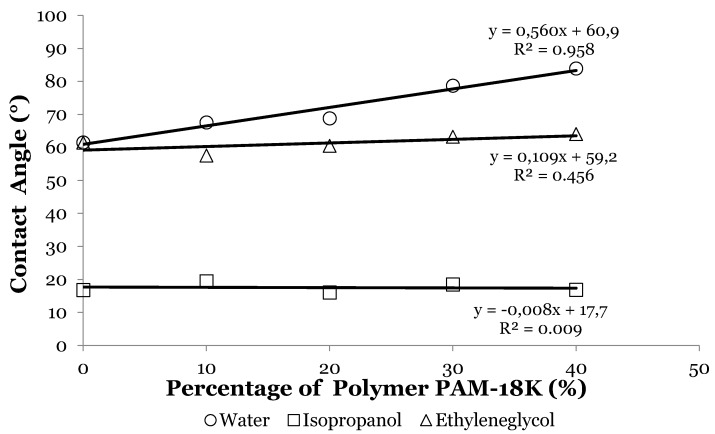
Variation of *θ_c_* for ampicillin trihydrate-PAM-18K tablets respect to the three test liquids.

This behavior shows that the increase in the polymer percentage in the tablets leads to an increase of *θ_c_*, suggesting that the tablets’ surface becomes more hydrophobic [34]. The results of variation of *θ_c_* with respect to the different proportions of PAM-18K polymer using ultra-pure water and other pure liquids are shown in Figure 5.

These results show different behaviors of *θ_c_* in relation to the liquid used and the percentage of PAM-18K polymer in the tablets. In the case of the system formed by ampicillin trihydrate without polymer and with water as reference liquid, it was observed that *θ_c_* = 61.52°. This value suggests that a spreading and wetting phenomena between the water droplet on the tablet surface as *θ_c_* is less than 90°. Moreover and as mentioned above, increasing the amount of polymer sample in the tablets results in a gradual increase of *θ_c_* to a value of 83.93°. This value is very close to the limit value (*θ_c_* = 90°), at which it has been widely reported that no spreading or wettability occurs. This variation in *θ_c_* with the percentage of polymer in the tablets can be explained based on the chemical structure of the system components. In the case of ampicillin trihydrate, it is found to have a variety of polar functional groups that allow it to interact attractively with droplets of water, favoring the relaxation phenomenon. However, when the PAM-18K polymeric material is incorporated within the tablets the opposite effect occurs, where a lower spread degree on the tablet surface was observed. This result is very interesting and can be explained based on the chemical structure of the monomer unit of PAM-18K, which corresponds to potassium dicarboxylate attached to an alkyl chain (18 carbons olefin). In this regard, the polymeric material in the surface area acquires a specific orientation, where the alkyl chains are oriented towards the gas phase, while part of the carboxyl groups are oriented towards the interior of the tablet [63]. This particular orientation of the alkyl chains on the tablets surface means that the surface becomes more apolar, hydrophobic interactions with water droplets are present and thus a lesser degree of spreading is present and consequently there is an increase in the contact angle [32].

In order to provide further support for this hypothesis, two pure less polar liquids compared to water and which polarity decreases gradually were evaluated. These liquids were ethylene glycol and isopropanol, respectively [49]. The results of *θ_c_* obtained for the model drug tablets at different proportions of PAM-18K polymer and using three test liquids are described in Figure 5, which shows that both ethylene glycol and isopropanol display a different behavior from that shown by water. It is observed that there is not a gradual increase in *θ_c_* with the amount of PAM-18K polymer, but rather it tends to remain constant, and the slopes for the linear fit are closer to zero value. These results can be explained based on the polar and dispersive contributions to the surface tension as well as the dielectric constant values of the liquids used (Table 2).

For both isopropanol and ethylene glycol there is a tendency of *θ*_c_ to remain constant regardless of the amount of polymer in the tablets, indicating that there exist attractive interactions between the tablets’ surface and the organic liquid, either through dispersive Van der Waals interactions or polar interactions by hydrogen bonding. Nevertheless, in the case of water a balance between attractive and repulsive interactions takes place, where the repulsive interaction increases strongly with the amount of polymer in the tablet, affecting considerably the surface free energies and water absorption rates in the compressed matrices.

It is important to highlight that a good measure of contact angle requires a characterization of the surface degree of roughness, as this parameter can affect the overall uncertainties in each measurement. However, in our case, the preparation of excessively porous surfaces presented an experimental problem at the moment of carrying out the hysteresis analysis because of the quick absorption of the liquid and also the drop penetration into the tablets. Currently, we are developing a specific study in order to evaluate the relationship between surface texture, rugosity and drug release in solid matrix systems with similar polymeric materials.

### 4.6. Determination of Surface Free Energy (SFE)

The work of adhesion (*W_adh_*) was determined by the Young-Dupre model, while the surface free energies (SFE) were determined from the Neumann and OWRK models, respectively. The results are summarized in Table 3.

All models evaluated show a decrease in the SFE of the tablets with the increase of PAM-18K polymer proportion in the matrix. This result suggests that the interface becomes more apolar in the presence of polymer. In the case of the OWRK model which is able to predict the SFE in dispersive and polar components very interesting results are observed with increasing polymer in the tablets. It can be seen that by increasing the amount of PAM-18K, the dispersive contribution to the SFE increases, while the polar contribution decreases, indicating that indeed the tablet surface becomes more apolar with an increased amount of polymer. This effect allows the tablet surface to interact differently with the various test liquids. In the case of water, a balance between attractive interactions and hydrophobic repulsion occurs, while organic liquids as ethylene glycol and isopropanol show attractive interactions taking place through Van der Waals interactions [50].

### 4.7. Determination of Water Absorption Rate on the Tablets Surface

**Figure 6:**
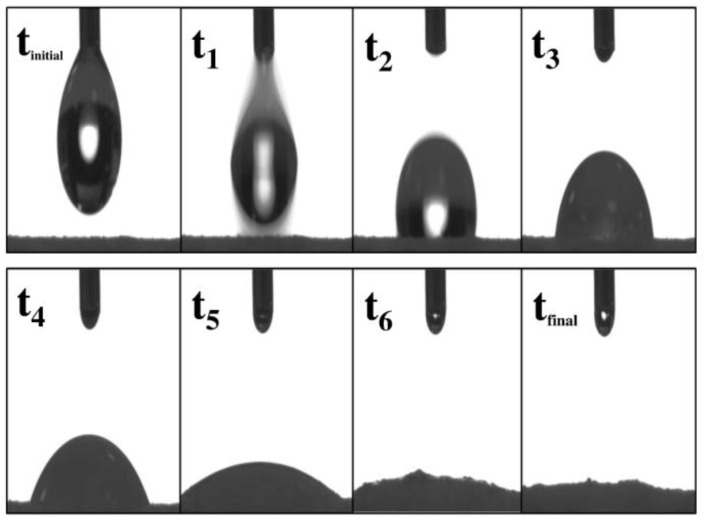
Water absorption process on surface of tablets at different times.

In the case of porous surfaces such as those formed by pharmaceutical dosage systems like tablets, it is very important to know the absorption rate of the liquid into the tablets, since depending on this phenomenon, the effects of the drug release in a particular dissolution medium could be conditioned. This water absorption phenomenon can be observed through the initial fall of the drop on the tablets’ surface, which progressively occurs with a variation of the liquid spreading combined with a water absorption effect, as shown in Figure 6.

**Figure 7:**
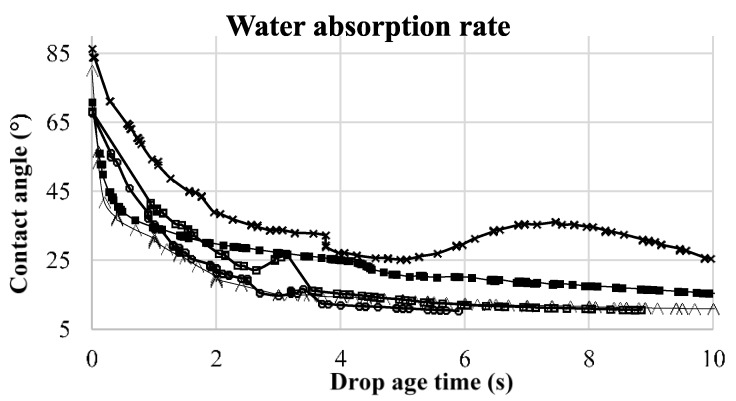
Ultra-pure water absorption profiles on the solid surface of ampicillin and PAM-18K tablets versus time. ■ = 0%, ○ = 10%, □: 20%, ×: 30%, ∆: 40%.

This phenomenon leads to a change in the contact angle versus time, which will depend on the particular characteristics of each surface In Figure 7, the absorption profiles of ultra-pure water are shown for ampicillin trihydrate and PAM-18K tablets containing different proportions of polymer For this, a variation profile of *θ_c_* as a function of elapsed time, defined as drop age, was established [63,66].

It can be seen that the absorption profiles of water on the tablet surfaces change depending on the amount of polymer on the tablets. In the case of ampicillin trihydrate tablets without PAM-18K polymer, it is seen that the change in *θ_c_* is reached in less time, while in tablets with polymer, this change of *θ_c_* requires more time.

This result suggests that the effect of spreading and water absorbance are conditioned by the amount of polymer on the surface, which is very consistent with previously observed results. In order to establish a rate parameter associated with this absorption phenomenon, a difference between initial *θ_c_* and final *θ_c_* with respect to the total time of drop age determined by the instrument was established. The results are summarized in Table 4.

Table 4, show that increasing the polymer amount within the tablet leads to an increase in the water absorption time, from 5.90 s to 15.80 s, whereby the absorption rate is delayed by the amount of PAM-18K. In the case of the tablets without polymer, the absorption time and the absorption rate are higher and smaller, respectively, than when the polymer is present. This effect is a consequence of the high degree of compaction of ampicillin trihydrate which influences the tortuosity of compressed tablets [13,67]. Such a result is very interesting when analyzed together with the results previously obtained in the tablet disintegration studies, where a similar trend is observed in the disintegration time, which increases with increased amount of polymer within the tablet (10%–40%). Those results reinforce our hypothesis that the PAM-18K polymer changes the polarity of the tablet surface, turning it more hydrophobic and modulating the thermodynamic behavior. In order to provide a better understanding of these phenomena a kinetic study of the model drug release was conducted in two in vitro dissolution media, as described below.

### 4.8. In vitro Dissolution Tests of the Model Drug

**Figure 8:**
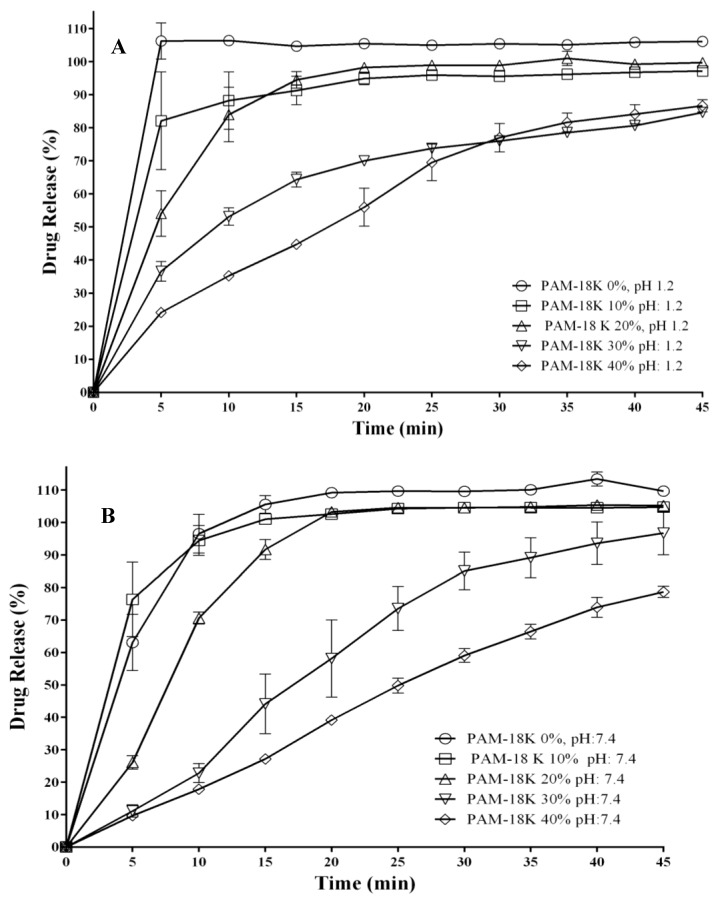
Dissolution profiles of ampicillin trihydrate from compressed tablets containing different proportions of PAM-18K polymer in two in vitro dissolution media with ionic strength of 0.15 M: (**A**) pH 1.2; (**B**) pH 7.4.

The results of the dissolution profiles for each ampicillin trihydrate tablet containing different proportions of PAM-18K polymer in two in vitro dissolution media under simulated physiological conditions are shown in Figure 8.

Figure 8 shows that the increase in percentage of polymer within the tablet leads to a change in the dissolution profile of the model drug in both in vitro dissolution media. In the case of tablets with percentages of polymer between 0% and 20%, a quick dissolution rate is observed, in a similar way to immediate release tablets, while for tablets with percentages of polymer between 30% and 40% a marked change is observed in the release profile [56,68]. These results are consistent with those observed in disintegration and surface property studies of the tablets where increasing the PAM-18K polymer levels in the tablets modify such properties. In relation to each dissolution profile, the values corresponding to the time when the maximum drug dissolution was achieved are summarized in Table 5.

It is noteworthy that although the statistical analysis carried out for the values of the dissolution efficiency only showed significant differences for the case of a polymer percentage of 40% in the dissolution medium at pH 1.2, it is possible to see a decreasing trend in that parameter with increasing amount of the PAM-18K polymer within the tablet. In the case of tablets with rates between 0% and 20% of polymer, rapid dissolution of ampicillin trihydrate occurs at both pH values, while for tablets with percentages between 30% and 40% of polymer, there is a decrease in the dissolution efficiency, which is more affected at pH 7.4 than at pH 1.2 [56]. In an effort to provide an explanation for these results and relate them to a possible release mechanism, several semi-empirical kinetic models were evaluated.

### 4.9. Kinetic Study of the Model Drug Release

For this study the zero order, first order, Higuchi and Korsmeyer-Peppas models were taken into account. The latter was only used for tablets with PAM-18K polymer in percentages of 30% and 40%, due to the fact that at lower proportions the release was very fast. The results obtained in the evaluation of these kinetic models are summarized in Table 6.

In the case of tablets tested in dissolution medium of pH 1.2, it is observed that data fits well to the order one and/or Higuchi models, suggesting that the drug release from the solid matrix is a Fickian type process, and it is favored by the gradient of chemical potential between the drug in the tablet and the dissolution medium [58,69,70]. This result is consistent since ampicillin trihydrate is a soluble drug in an aqueous medium, even at that pH. In the case of tablets tested in dissolution medium of pH 7.4 with percentages of polymer between 0% and 20%, similar behavior was also observed, as mentioned before, however in the case of tablets with percentages of polymer between 30% and 40% it was found that the data fit better the Korsmeyer-Peppas and zero order models, suggesting that the release mechanism is controlled by the process of polymer chain relaxation, where the dissolution medium penetrates the compressed matrix, forming pores and then eroding it [31,59]. These results are very interesting and moreover, if related to studies of disintegration and dissolution. In the disintegration studies it was observed that increasing the amount of polymer in the compressed matrix leads to an increase in the disintegration time of the tablets. Furthermore, in the dissolution studies it was found that the increase of polymer in the compressed matrix leads to a decrease of the dissolution rate of the drug [70]. Both results are related to the consistency or form that the tablets kept during these processes. In the case of those tablets with polymer percentages between 0% and 20%, it was observed that the disintegration times were much lower than when the tablets had higher percentages of polymer, i.e., 30% and 40%. For dissolution studies a similar effect was observed where tablets disintegrated completely releasing the drug into the dissolution medium almost immediately, while for proportions of polymer between 30% and 40%, the tablets do not disintegrate completely, remaining part of the core. Hence, the PAM-18K polymer amount in the compressed matrix leads to different degrees of hydrophobicity on the tablets’ surface, thereby the mode of interaction with the dissolution medium will be different and thus the PAM-18K polymer controls the release of the model drug in the evaluated dissolution media [56].

## 5. Conclusions

Increasing the amount of amphiphilic PAM-18K polymer within ampicillin trihydrate tablets leads to an increase in the hardness and disintegration time of the resulting tablets. In the case of tablets with PAM-18K polymer at 10% and 20%, disintegration times are lower than when the polymer is 30% to 40%. Also, the thermal analysis evidenced a strong interaction between the solid blends of polymeric materials and the ampicillin drug, which becomes greater with increasing polymer in the mixture. Moreover, studies of surface thermodynamic properties showed that an increase of the PAM-18K polymer in tablets led to an increased contact angle *θ_c_* and a decrease in the surface free energy, indicating that the tablets’ surface becomes more hydrophobic. It is important to highlight that a correlation between all the parameters associated with contact angles and the surface roughness must be performed in futures studies, because the porosity in the solid matrix could lead and affect the liquid absorption contact angle. The degree of hydrophobicity on the surface of the tablet influences the release mechanism of the model drug from the tablet to the dissolution medium. In the case where the proportion of polymer in the tablet is 10% to 20%, the drug release is controlled by a Fickian diffusion processes, while when the percentages of polymer are 30% and 40%, the release mechanism becomes a controlled release process driven by swelling and erosion of the tablet. Thus, the amount of PAM-18K polymer in the tablets can modulate the release of a very soluble drug in aqueous media by variation of the release mechanism, from immediate release to an extended release.

## Figures and Tables

**Table 1:** Results of hardness and disintegration time for ampicillin trihydrate tablets at different proportions of PAM-18K polymer and different compression forces.

% PAM-18K in the Tablet	Applied Pressure (psi)	Average Hardness (kp)	Disintegration Time (min:s)
0	200	4.48 ± 0.24	3:45
	300	7.86 ± 0.71	4:35
	400	9.51 ± 0.57	8:30
10	200	6.15 ± 0.72	2:48
	300	8.91 ± 0.71	3:26
	400	12.46 ± 1.45	3:28
20	200	7.83 ± 0.69	3:32
	300	10.17 ± 1.68	3:33
	400	12.99 ± 2.10	5:23
30	200	12.54 ± 0.34	4:20
	300	13.74 ± 0.52	7:12
	400	17.34 ± 0.36	9:00
40	200	15.67± 1.13	9:50
	300	18.75± 0.30	13:51
	400	Undetermined	17:30

**Table 2:** Characterization values for the surface tension of the liquids used in the test of contact angle values (*θ*_c_) for ampicillin trihydrate-PAM-18K.

Physical Property		Water	Ethylene Glycol	Isopropanol
* Surface tension (mN/m)	Total	72.1	48.0	23.0
	Dispersion contribution	19.9	29.0	19.5
	Polar contribution	52.2	19.0	3.5
** Dielectric constant		80.1	68.0	17.9

* Values taken from Birdi and Ohm [64]; ** from Lippold, Busscher et al. [65].

**Table 3:** Determination of SFE for ampicillin trihydrate and PAM-18K tablets.

% PAM-18K within the Tablet	Surface Free Energy (mJ/m^2^)						
	Young-Dupre	Neuman (EdE)	Owens, Wendt, Rabel & Käelble (OWRK)				
	(*W_adh_*)		Total SFE	Dispersive	Polar	SFE (R^2^)	SFE (s)
0%	106.5 ± 2.8	46.4 ± 1.5	39.1 ± 2.4	5.1	33.9	0.970	4.1
10%	99.6 ± 0.7	42.7 ± 0.4	34.3 ± 0.4	7.9	26.4	0.998	0.8
20%	98.2 ± 1.6	41.9 ± 0.8	33.0 ± 1.1	8.1	24.9	0.994	1.6
30%	86.2 ± 3.3	35.8 ± 1.7	26.5 ± 1.3	11.6	14.9	0.997	0.9
40%	79.7 ± 3.1	32.5 ± 1.5	24.5 ± 0.6	14.3	10.3	0.999	0.4

* R^2^ is the linear determination coefficient for SFE by the OWRK method; s: the standard deviation for SFE in the OWRK model.

**Table 4:** Results of absorption rate on the compressed matrices.

% PAM-18K within the Tablet	Initial *θ_c_* (°)	Final *θ_c_* (°)	Absorption Time (s)	Absorption Rate (°/s)
0	61.9	10.0	16.9	3.2
10	72.7	10.3	5.9	10.6
20	86.2	10.7	8,8	8.5
30	86.4	25.5	9.9	6.1
40	80.5	10.3	15.8	4.4

**Table 5:** Values of maximum dissolution time and the dissolution efficiency (DE) in vitro for compressed tablets of ampicillin trihydrate for different proportions of PAM-18K at 37.0 °C.

pH of the Medium	% Polymer in the Tablet	% of Dissolved Drug	Dissolution Time (min)	Dissolution Efficiency
1.2	0	100	25	99.64
	10	100	25	87.69
	20	100	25	86.49
	30	84	45	63.91
	40	84	45	57.29
7.4	0	100	15	96.89
	10	100	15	93.87
	20	100	20	84.84
	30	96.8	45	58.42
	40 *	78.7	45	42.49

The maximum dissolution time tested was 45 min. * *p* < 0.05.

**Table 6:** Parameters and coefficients for the determination of various kinetic models of dissolution.

pH of the Medium	% Polymer in the Tablet	Zero Order		First Order		Higuchi		Korsmeyer-Peppas		
		*k_0_*	R^2^	*k_1_*	R^2^	*k_H_*	R^2^	*k_r_*	N	R^2^
1.2	0	2.978	0.558	0.050	0.867	18.655	0.924	-	-	-
	10	1.148	0.271	0.022	0.273	11.754	0.932	-	-	-
	20	3.642	0.767	0.082	0.992	23.110	0.978	-	-	-
	30	1.497	0.758	0.016	0.933	12.244	0.949	2.542	1.040	0.822
	40	1.868	0.933	0.020	0.991	14.006	0.982	1.399	1.340	0.932
7.4	0	6.421	0.795	0.132	0.998	27.091	0.964	-	-	-
	10	7.086	0.894	0.146	0.947	28.270	0.991	-	-	-
	20	5.444	0.959	0.099	0.919	24.300	0.933	-	-	-
	30	2.325	0.953	0.033	0.963	16.843	0.936	1.063	1.360	0.996
	40	1.826	0.993	0.015	0.981	12.902	0.928	1.135	1.184	0.993

* (-) model not evaluated due to insufficient data.
